# Research topics in occupational medicine, 1990–2022: A text-mining-applied bibliometric study

**DOI:** 10.5271/sjweh.4177

**Published:** 2024-10-01

**Authors:** Kosuke Sakai, Tomohisa Nagata, Takahiro Mori, Shunsuke Inoue, Hideki Fujiwara, Kiminori Odagami, Nuri Purwito Adi, Masayuki Tatemichi, Koji Mori

**Affiliations:** 1 Department of Occupational Health Practice and Management, Institute of Industrial Ecological Sciences, University of Occupational and Environmental Health, Kitakyushu, Japan.; 2 Department of Preventive Medicine, Tokai University School of Medicine, Isehara, Japan.; 3 Department of Community Medicine, Faculty of Medicine, Universitas Indonesia, Jakarta, Indonesia.

**Keywords:** bibliographic study, bibliometric analysis, industrial medicine, text mining

## Abstract

**Objective:**

Occupational health has been influenced by societal and industrial changes. This study aimed to clarify topic trends in occupational health research in 1990–2022.

**Methods:**

We conducted a text-mining-adjusted bibliometric study using research titles in occupational health. Data on research titles and the years of publication were collected from 26 peer-reviewed journals on PubMed. Using morphological and correspondence analyses in text mining, we structured research topics into multiple categories and visualized the relationship between all categories and publication years. Statistical analyses were conducted using the text mining software – KH Coder 3.0.

**Results:**

We obtained 48 645 articles containing 714 890 words in their titles. The research topics were classified into 4 categories and 17 subcategories, of which those of occupations; countries; non-intervention; psychosocial factors; lifestyle factors; safety; symptoms; therapy and care; and productivity have recently shown an increasing trend. In contrast, the subcategories of risk, chemical factors, disease, and organ damage showed decreasing trends. Chemical factors, which were the main topics in the 1990s, included risk, organ damage, and disease. Productivity, the main topic in the 2020s, co-occurred with lifestyle factors, symptoms, and intervention.

**Conclusions:**

Focal areas in occupational-health research shift according to societal trends. Occupational-health research has primarily analyzed issues in developed countries with capitalist values and may not have sufficiently covered issues in developing countries. It is imperative for policymakers and public funding bodies to determine priorities for investigation in the field.

Occupational health has been shaped by work, social evolution, the changing modes of production, shifting economic powers, and demographic changes in the workforce (1). Currently, new studies are emerging, such as those on new toxic substances known as nanomaterials, new employment, such as precarious work, and health problems associated with teleworking during the COVID-19 pandemic (2–4). Individuals' work is identified as a determinant of health, encompassing challenges, such as technological development, strong associations with individuals' socioeconomic status, the growth of migrant and precarious workers, prolonged and irregular work hours, and the impacts of climate change (5). As workers in many developed countries are aging, there is a focus on interventions at various levels – including policy, work environment, and personal attributes – to ensure continuity in the labor market throughout their lifetimes (6).

Occupational health research has reported various changes in the covered topics. According to previous studies that analyzed the topics of occupational health, the 1980s saw an increase in epidemiological methods; the 1990s, occupational cancer and musculoskeletal pain research; and the 2000s, work-life balance and worker productivity research (7, 8). An occupational health journal that has been published for over 50 years reported that topics in the field have changed from chemical exposure in 1975–2004 to psychosocial work environment, shift work, and physical workload in 2015–2023, with higher references to mental disorders and musculoskeletal disorders (9). Understanding how topics addressed in occupational-health research have changed will give a clearer picture of the relationship between occupational health and society. This will help scientists consider topics that should be explored in the field in the future. In addition, it will help governments and research foundations consider topics in which investments should be made.

Previous studies that deal with the topics in occupational health research have analyzed keywords and research fields of articles (10, 11). However, the targeted articles were limited to a specific journal, field, or the time periods of publication (7, 8, 11, 12). For a comprehensive understanding of research trends in occupational health, a large number of journals over long periods should be surveyed. With an advancement in computing power, text mining has been utilized in medical research (13, 14). We can identify research trends by analyzing the text data used in the title of an article as it describes the topic of the research (15). To understand the diversity of the research topic, it would be better to analyze titles that are not restricted in expression versus keywords, which are often selected from a limited set of words in a corpus. Further, titles are more useful than keywords in allowing researchers to consider the context in which words are used. By utilizing text mining in bibliometric studies, we can analyze many articles simultaneously.

This study aimed to identify changes in topics in the field of occupational health research using a text-mining method. Taking a chronological view of the topics addressed by occupational health research, we aimed to reveal the relationship between occupational health and society and discuss the future in which occupational health will progress.

## Methods

### Study design

We conducted a bibliographic study using text mining to analyze research articles that deal with occupational health. Because the title of a research article expresses the main theme of the paper, our analysis targeted text data in titles. We identified chronological changes in research topics on occupational health by exploring and structuring words used in titles.

### Data collection

**Figure 1:**
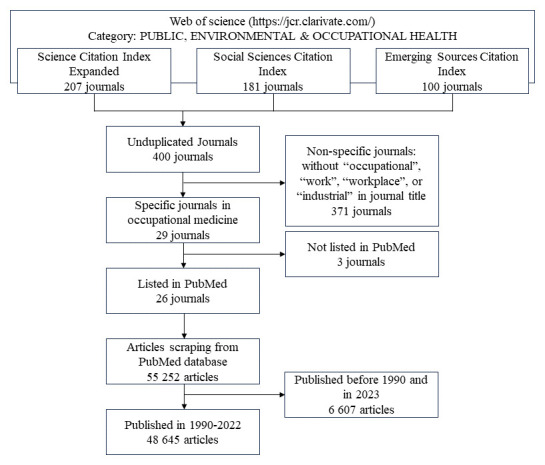
Flow diagram of selection procress for articles

Figure 1 is a flow diagram that describes the data-collection process. We identified occupational health journals, selected articles, and obtained detailed information. We identified journals categorized as “PUBLIC, ENVIRONMENTAL, and OCCUPATIONAL HEALTH” in the Web of Science (jcr.clarivate.com). The Science Citation Index Expanded included 207 journals in the natural sciences; the Social Sciences Citation Index included 181 journals in the social sciences; and the Emerging Sources Citation Index included 100 journals with a focus on specific areas of interest, which is expected to expand in the future. We eliminated journals with matching journal names and identified 400 journals.

To include journals with occupational health as the main theme, we used the inclusion criteria for journals with the following words in their names: “occupational,” “work,” “workplace,” and “industrial.” We identified 29 out of 400 journals. Further, to obtain consistent journal information, we excluded three journals that had not been published in PubMed: *Cadernos Brasileiros de Terapia Ocupacional* (the *Brazilian Journal of Occupational Therapy*), the *Journal of Health and Safety at Work*, and the *Journal of Clinical Social Work and Health Interventions*. Using data scraping, we extracted the titles and publication years of 55 252 articles from 26 journals in PubMed (7 November 2023). To collect sufficient data – a total of 5 000 words in every publication year – we excluded articles in 1958–1989. Finally, we created a database of 48 645 articles on occupational health published between 1990–2022, after excluding 6 607 articles published before 1990 or in 2023, when the data collection was carried out.

### Statistical analysis

The study was conducted in four steps. First, morphological analysis was applied to the titles of occupational-health articles to identify frequent words. Second, a correspondence analysis between frequent words and publication years was developed. Third, the topic of occupational-health research was structured into categories with coding rules for frequent words and their titles. Fourth, reference rates were chronologically determined for each topic.

### Morphological analysis

Through morphological analysis, a text is divided into its smallest meaningful linguistic units and its parts of speech and grammatical characteristics are identified. A morphological analysis was performed on the titles of all articles. Words beginning with the upper case used in the titles were changed to the lower case for data cleaning. Words suggesting countries, such as the United States and India, were specified as mandatory extraction words. A Stanford POS Tagger was used as the dictionary for text mining. We used the results of the morphological analysis to create a list of words in the order of the frequency of occurrence.

### Correspondence analysis

Correspondence analysis is a dimensional reduction technique used in text mining to visualize the relationship between words and external variables. Words that are far removed from the origin and whose directions are closer to an external variable are considered more characteristic of that variable. To understand the trend of the frequent word occurrence over time, we conducted a correspondence analysis (16). To perform correspondence analysis with words that are easy to interpret, we extracted nouns after excluding verbs, adjectives, conjunctions, interrogatives, prepositions, and proper nouns. We also summarized the publication years into seven groups as external variables: 1990–1994, 1995–1999, 2000–2004, 2005–2009, 2010–2014, 2015–2019, and 2020–2022. Then, we visualized the relationship between the top 100 most frequent nouns and publication-year groups using correspondence analysis. By observing the results of the correspondence analysis, we discussed changes in the topics of occupational-health research over time, which provided us with clues for structuring the next phase of the analysis.

### Structured topics in occupational health research

Based on the results of morphological and correspondence analyses, we structured the topics of occupational-health research into several categories and subcategories and created coding rules for each subcategory. According to the coding rules, a computer can determine subcategories that are listed for each title. To form coding rules, we selected 300 most frequent words and categorized them through the reading of actual titles to confirm contexts in which all words were used. We excluded words that were used in any context, such as work, from our coding rules. The three authors discussed improving objectivity in the creation of coding rules. Following the coding rules, we analyzed all titles to calculate the percentage of mentions in each subcategory.

### Topic changes over time

We analyzed all titles based on coding rules and calculated the percentage of references in each category. We used a heat map to visualize how mentions in each category changed over time. We used two indices percentages and Pearson's residuals – in the heat map. Percentages were calculated using the number of articles for each year as the denominator and articles mentioning each subcategory as the numerator. Pearson residuals were used in the heatmap analysis. This indicates the degree to which the observed values deviate from the expected values in the analysis of mentions in a particular category. Pearson residual = (O−E)/√E, O is the observational number of titles; E is the totally expected number of titles of all publications in the year. When the mentions of a particular category are more frequent or rarer than expected, they can be represented as gray intensity on the heatmap (17). Additionally, we created a similarity matrix to demonstrate the co-occurrence between subcategories. We calculated Jaccard values for combinations in all subcategories as a measure of co-occurrence (18). The Jaccard value between A and B was calculated as the number of titles containing A and B, divided by the number of titles containing A or B. The numbers indicate the strength of the relationships between subcategories in the titles.

For the statistical analysis, we used the text mining software KH Coder 3.0, which was engineered by Higuchi of Ritsumeikan University, Kyoto, Japan (khcoder.net) (19). The application of the software in text-mining studies on public health motivated us to select it (20, 21). We used R statistical software to support our analysis. To detect the characteristics of the articles in detail, we calculated the number of words in each article using Microsoft Excel.

## Results

### Characteristics of articles

Table 1 presents the number of articles by publication years and journals. We collected 48 645 articles, more than half of which were published between 2010–2022. The average number of words in the title increased over time from 10.7 words in 1990–1994 to 14.6 words in 2020–2022. The order of journals with the most articles is as follows: the Journal of Occupational Environment Medicine with 5 471 articles; Work with 5 445 articles; and American Journal of Industrial Medicine with 4 772 articles. Of 26 journals, eight were in publication as of 1990.

**Table 1:** Characteristics of the research articles. SD=standard deviation.

		Article			Words in title	
		N	%		Mean	SD
**Total**		48 645	100		12.9	5.0
**Publication year**						
	1990–1994	3 104	6.4		10.7	4.9
	1995–2009	5 435	11.2		11.6	4.7
	2000–2004	5 516	11.3		12.1	4.8
	2005–2009	6 974	14.3		12.4	4.8
	2010–2014	10 066	20.7		12.9	4.7
	2015–2019	10 454	21.5		13.7	5.0
	2020–2022	7 096	14.6		14.6	5.0
**Journals, publication year**						
	J Occup Environ Med, 1995–2022	5 471	11.2		13.6	4.9
	Work, 1990–2022	5 445	11.2		12.7	4.9
	Am J Ind Med, 1990–2022	4 772	9.8		12.3	4.5
	Occup Environ Med, 1994–2022	4 569	9.4		13.4	4.9
	Occup Med (Lond), 1992–2022	3 548	7.3		9.1	3.8
	Int Arch Occup Environ Health, 1990–2022	3 270	6.7		14.3	5.0
	Scand J Work Environ Health, 1990–2022	2 549	5.2		12.5	4.8
	Toxicol Ind Health, 1990–2022	2 335	4.8		13.5	5.0
	Ind Health, 1990–2022	2 008	4.1		13.6	4.7
	J Occup Environ Hyg, 2004–2022	1 981	4.1		13.1	4.9
	Int J Occup Saf Ergon, 1995–2022	1 646	3.4		13.4	4.6
	Int J Occup Med Environ Health, 1994–2022	1 554	3.2		13.9	5.7
	J Occup Health, 2003–2022	1 400	2.9		14.5	4.8
	Arh Hig Rada Toksikol, 1990–2022	1 264	2.6		11.7	4.7
	New Solut, 1990–2022	1 209	2.5		10.3	6.0
	J Occup Health Psychol, 1996–2022	938	1.9		13.6	4.0
	Arch Environ Occup Health, 2005–2022	848	1.7		12.5	4.7
	Soc Work Public Health, 2007–2022	751	1.5		13.8	4.4
	Saf Health Work, 2010–2022	714	1.5		13.8	4.2
	Ann Work Expo Health, 2017–2022	676	1.4		14.2	4.7
	J Occup Med Toxicol, 2006–2022	610	1.3		14.8	4.9
	Indian J Occup Environ Med, 2007–2022	563	1.2		12.1	5.0
	Ann Occup Environ Med, 2013–2022	474	1.0		15.2	5.2
	Occup Health Sci, 2017–2022	30	0.1		13.8	3.6
	J Workplace Behav Health, 2009–2019	13	0.0		12.7	3.9
	Int J Workplace Health Manag, 2010–2021	7	0.0		11.6	4.0

### Morphological analysis

The morphological analysis revealed that the titles of 48 645 articles contained 714 890 words. The supplementary material (www.sjweh.fi/article/4177), table S1, shows the top 40 most frequent words, the parts of speech, and the number of occurrences. 'Worker' occurred 7 975 times as a noun, 'occupational' 6 913 times as an adjective, and 'health' 6 811 times as a noun.

### Correspondence analysis

**Figure 2:**
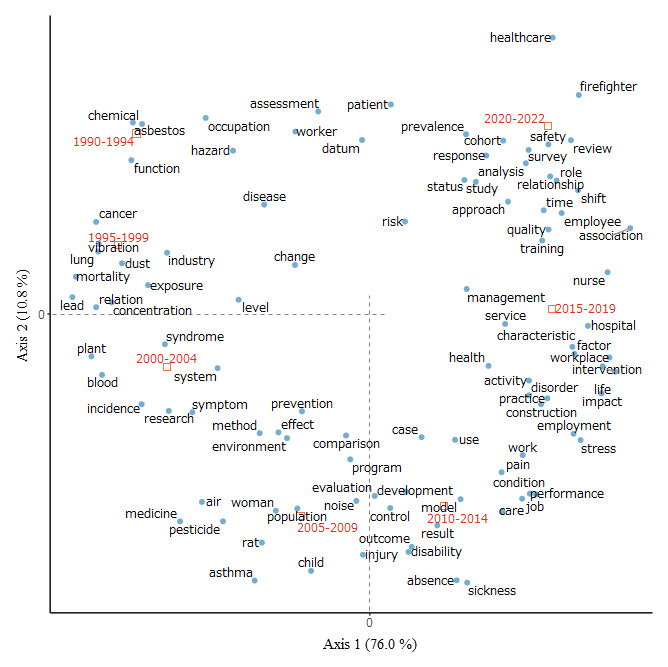
Correspondence analysis of top 100 nouns and publication years.

Figure 2 shows the results of the correspondence analysis. The following terms were used: chemical, asbestos, and function in 1990–1994; vibration, cancer, and lung in 1995–1999; plant, blood, and incidence in 2000–2004; rat, asthma, and child in 2005–2009; sickness, absence, and result in 2010–2014; hospital, nurse, and service in 2015–2019; and healthcare, firefighter, and safety in 2020–2022. The results also indicate that the first two components accounted for a significant portion of variability. Specifically, axis 1 explained 76.0% of variability, whereas axis 2 accounted for 10.8%. Together, these components explained much of the phenomenon, accounting for 86.8% of total variability.

### Structured topics in occupational health research

We structured the topics of occupational health research into 4 categories and 17 subcategories, as table 2 shows. For example, articles containing words like 'workplace', 'industry', or 'medical employee' in the title were classified as referring to the subcategory of the occupations. After adjusting the coding rule for all articles, 25.0% of the articles referred to the occupations, 22.0% to the risk, and 19.5% to the non-intervention. However, 15.0% did not refer to any subcategories.

**Table 2:** Structure of title contents and results of cross tabulation following coding rules

Category	Subcategory	Words	N	%
Background	Occupations	workplace, industry, medical, occupation, nurse, hospital, healthcare, firefighter, construction, plant, physician, service, driver, manufacturing, industrial, office, miner, company, coal, public, production, agricultural, farmer, home, school, student, professional	12 164	25.0
	Countries	Japanese, Japan, Unite States, Korea, India, Swedish	1 799	3.7
Study design	Non-intervention	cohort, analysis, review, incidence, survey, prevalence, systematic, longitudinal, case-control, cross-sectional, research, follow-up, meta-analysis, surveillance, questionnaire, self-reported, process	9 463	19.5
	Intervention	program, intervention, trial, controlled, approach, pilot, training, strategy, implication, tool	4 138	8.5
Exposure	Risk	exposure, risk, hazard, protective, protection, affect	10 712	22.0
	Chemical factors	air, asbestos, dust, lead, pesticide, chemical, pollution, acid, organic, metal, carbon, silica, particle, toxicity, oxidative, cadmium, airborne	6 652	13.7
	Psychosocial factors	stress, shift, psychosocial, psychological, strain, social, support, demand	4 588	9.4
	Lifestyle factors	activity, sleep, hour, behavior, smoking, life, body	3 190	6.6
	Physical factors	physical, vibration, field, noise, heat, radiation	2 951	6.1
	Biological factors	COVID-19, blood, pandemic, biological	1 845	3.8
	Ergonomic factors	ergonomic, ergonomics, computer	1 090	2.2
Outcome	Therapy and care	mortality, disorder, care, disability, management, prevention, compensation, ability, treatment, return, rehabilitation, emergency, education	7 168	14.7
	Organ damage	lung, respiratory, musculoskeletal, cardiovascular, heart, pulmonary, urinary, biomarker, cell, upper, skin, hand, damage, liver	6 634	13.6
	Disease	cancer, disease, asthma, depression, mesothelioma, illness, tunnel, carpal	5 407	11.1
	Symptoms	pain, symptom, mental, chronic, syndrome, back, fatigue, burnout, hearing	5 386	11.1
	Productivity	absence, sickness, cost, productivity, loss, performance, quality, effectiveness, well-being, task, satisfaction	4 020	8.3
	Safety	injury, safety	3 063	6.3
No coding*			7 314	15.0

*Response descriptions that did not fall into any subcategories.

### Topic changes over time

**Figure 3:**
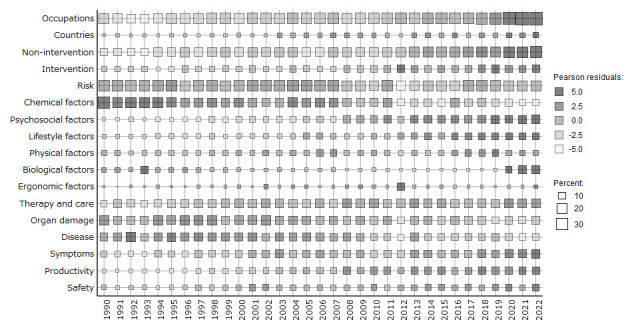
References to subcategories over time.

Figure 3 shows a heat map of the distribution of 17 subcategories from 1990–2022. During this period, the Pearson residuals for occupations, countries, non-intervention, psychosocial factors, lifestyle factors, safety, symptoms, therapy and care, and productivity show an increasing trend. In contrast, the Pearson residuals for risk, chemical factors, disease, and organ damage showed a decreasing trend. The Pearson residuals for the biological factors showed high values from 2020 to 2022.

Supplementary table S2 shows the co-occurrences of these subcategories. Relationships among all subcategories, in order of ascending Jaccard, were 0.17 for risk and chemical factors, 0.15 for occupations and non-intervention, 0.13 for occupations and therapy and care, 0.13 for non-intervention and disease, 0.13 for risk and organ damage, 0.13 for organ damage and disease.

## Discussion

Our text-mining analysis of the titles of the articles in occupational-health research from 1990 to 2022 revealed four categories and 17 subcategories. We identified five insights into research trends in the field.

In the 1990s, the predominant focus was on chemical factors (lead and asbestos), diseases (cancer and disease), and organ damage (lung and respiratory) (figures 2 and 3). These subcategories had high co-occurrence, as shown in supplementary table S2.

From 2000–2022, there was a shift towards psychosocial factors (stress and social), symptoms (pain and mental), and therapy and care (disability and care), as shown in figures 2 and 3. Supplementary table S2 indicates a high correlation between the subcategories.

From 2010–2022, productivity emerged as a significant topic, with related terms, such as sickness and performance (figure 2).

From 2020–2022, biological factors gained prominence, driven by a rise in COVID-19-related research, on which 12.6% articles focused in 2022 (figure 3).

Over the past three decades, there has been an increasing tendency of article titles mentioning the study design, spanning both the intervention and non-intervention subcategories (figure 3).

Chemical factors, diseases, and organ damage were the main topic in the 1990s in occupational health, which has been extensively studied since the Industrial Revolution. The International Labor Organization listed anthrax, lead, and mercury poisoning as workers' compensation conventions in 1920 and has attempted to focus on combating occupational diseases for many years (22). A previous study similarly reported that cancer was the health outcome of considerable interest in 1975–1984 (9). Under the influence of history, in the 1990s, many papers focused on chemical factors and the relationship between occupational diseases and organ damages. There have been many cases of respiratory illnesses among workers handling chemicals in factories, and health problems have been reported among not only workers but also community residents (23, 24).

An increasing number of articles have recently addressed psychosocial factors, symptoms, and therapy and care. This trend is consistent with the results of previous studies. According to a systematic review of occupational safety and health research from 1990–2018, since 2000 an increasing number of articles have addressed occupational stress and mental health (8). The previous study also reported that topics on psychosocial work environment and mental disorders have increased since 2015 (9). Researchers have reported that the incidence of occupational diseases is decreasing worldwide (25, 26). However, the working environment is rapidly changing because of technological innovations. These changes influence workers' stress, satisfaction, and motivation (27). For example, online remote work and flexible work arrangements create an 'always on' work environment that can remove the distinction between work and life (28). In globalization, working with individuals from different cultural backgrounds can create stress in the form of misunderstandings and communication barriers (29). In an aging workforce, workers' symptoms of sustainable work and the need for therapy and care may be highlighted (30).

A growing number of studies have discussed productivity. In 2018, research priorities in the UK included economic evaluation, cost-effectiveness, and disability management (31). It has also been reported that safety culture, safety atmosphere, sickness absence, and safety performance have been popular topics since 2010 (8). These studies support the consistency of the results of this study. From a social perspective, there is growing interest in corporate social responsibility (CSR) for stakeholders, including stockholders and investors, leading to an increase in occupational health research in the context of CSR (32). Productivity showed co-occurrence with lifestyle factors, symptoms, and interventions, which indicates that research focused on how productivity can be increased. Occupational-health research has expanded to cover not only the workplace environment but also workers' lifestyles.

Since 2020, the number of studies on biological factors has sharply increased. In addition to pandemics, wars, terrorism, and other disasters have quickly become topics in occupational health. Since the 9/11 attacks in the United States, much research has focused on firefighters (33).

References to the study design in titles have increased over the past three decades. A report published in 2001 recommended that titles contain information about the study design (34). In addition, reporting guidelines such as the STROBE statement recommend that the study design should be clearly stated in the title (35). Following these recommendations, the study design has been gradually described in the title. This would have been useful for readers to quickly find the literature they wanted to read given the huge number of scientific articles published.

The fact that a problem is no longer a research topic in occupational health does not imply that it has been resolved. Though warning about the risks of silica and other chemicals has been raised for decades, the issue has not yet been resolved (36). According to a systematic analysis of the WHO/ILO joint estimates of the work-related burden of disease and injury, the trachea, bronchus, and lung cancer were estimated to be the leading causes of work-related deaths after chronic obstructive pulmonary disease, ischemic heart disease, and stroke. The work-related burden of disease and injury was reported to be disproportionately high in the African region, South-East Asia region, and Western Pacific region (37). The incidence of tracheal, bronchial, and lung cancers attributable to occupational carcinogens is increasing in developing countries (38). With globalization, hazardous work that causes health problems for workers is increasing in developing countries where labor costs are lower and regulations regarding occupational health are less strict (39). Occupational health research has been conducted mainly in high-income countries (the United States, United Kingdom, and Sweden), with low- and middle-income countries falling behind (40). Work and mental health research has been conducted primarily in high-income countries, and workers in lower socioeconomic positions were overlooked (41). Systems in some developing countries are often inadequate to address exposure and its effects; access to hospitals may be limited; and necessary treatment may not be available. The findings of the study show that occupational-health research has been focusing on issues in high-income countries in the context of capitalism.

The strength of this study is that we collected a large amount of textual data – the titles of articles in occupational-health research – and used text-mining techniques to identify chronological trends in the field. The study could also determine which topics have been gradually addressed and how they relate to each other over the past 30 years. We found that major changes in society and industry influence occupational-health topics.

This study had some limitations. The first is the selection bias in the process of identifying articles on occupational health. Articles in the field of occupational health will also be published in journals that analyze public health. Owing to scraping technology, occupational health articles from these journals were excluded. In the future, when technology facilitates the collection of information on occupational health across search engines and journals, we can conduct more comprehensive bibliometric studies. The second limitation is our subjectivities, which influence the grouping of words in the correspondence analysis and the structuring of topics in occupational health research. Therefore, three researchers independently developed subcategories and coding rules to increase the reliability of this study. When they had different opinions on the classification, they discussed and decided on the classification. The third limitation relates to the influence of time on titling. We failed to adjust for the fact that the number of words in the title increased over time. The higher the average number of words in a title, the more references the subcategory had. An increase in mentions may not always mean that a topic is increasingly used as a research topic. We cannot clearly distinguish whether the mentions of a subcategory have recently increased because greater attention is paid to the subcategory or because of the increased number of words in each title. However, the average number of title words per journal varied from about nine to fifteen. This supports the importance of the large number of journals included in this study. The fourth limitation concerns the methodology in text-mining research. This novel method has not yet been adequately established. Our study is a small research project and we hope to provide a reference for validating the methodology in the future. Therefore, we carefully reviewed these methods.

### Concluding remarks

This study identified research topics in occupational health over the past 30 years. These results suggest that topics in occupational health are subject to changes in social majorities. Considering that precarious workers in developing countries may not be targeted in research, policymakers and those who allocate public funds should decide which topics should be investigated in occupational health.

## Supplementary material

Supplementary material

## Data Availability

Data used in this study are collected from an online open database. Please contact the corresponding authors for further details.
