# Supplementary material for: Research topics in occupational medicine, 1990–2022: A text-mining-applied bibliometric study
Source: Scand J Work Environ Health. 2024 Sep 30;50(7):567–76. doi: 10.5271/sjweh.4177 (PMC11479747; doi:10.5271/sjweh.4177)
Supplement: Supplementary material [file SJWEH-50-567-S001.pdf]

**Research topics in occupational medicine, 1990–2022: A text-mining-applied bibliometric study[1]**

Kosuke Sakai, MD,[2] Tomohisa Nagata, PhD, Takahiro Mori, PhD, Shunsuke Inoue, MD, Hideki Fujiwara, MD, Kiminori Odagami, MD, Nuri Purwito Adi, PhD, Masayuki Tatemichi, PhD, and Koji Mori, PhD

1. *Supplementary materials*
2. *Corresponding author: Kosuke Sakai, MD, MOH, Department of Occupational Health Practice and Management, Institute of Industrial Ecological Sciences, University of Occupational and Environmental Health, 1-1 Iseigaoka Yahatanishi-ku, Kitakyushu 807-8555, Japan [Email: kousuke-sakai0530@med.uoeh-u.ac.jp]*

Supplementary Table S1. Frequent word list extracted using text mining

| Frequent word list | | | | | | | |
|---|---|---|---|---|---|---|---|
| No. | Word | POS* | Frequency | No. | Word | POS* | Frequency |
| 1 | worker | Noun | 7975 | 21 | evaluation | Noun | 1424 |
| 2 | occupational | Adj† | 6913 | 22 | employee | Noun | 1383 |
| 3 | health | Noun | 6811 | 23 | review | Noun | 1337 |
| 4 | exposure | Noun | 6703 | 24 | industry | Noun | 1333 |
| 5 | study | Noun | 6036 | 25 | association | Noun | 1314 |
| 6 | work | Noun | 4708 | 26 | disorder | Noun | 1303 |
| 7 | effect | Noun | 3811 | 27 | expose | Verb | 1301 |
| 8 | risk | Noun | 3634 | 28 | use | Verb | 1275 |
| 9 | factor | Noun | 2341 | 29 | mortality | Noun | 1273 |
| 10 | cancer | Noun | 2162 | 30 | symptom | Noun | 1252 |
| 11 | assessment | Noun | 1813 | 31 | cohort | Noun | 1236 |
| 12 | workplace | Noun | 1737 | 32 | musculoskeletal | Adj† | 1226 |
| 13 | injury | Noun | 1715 | 33 | lung | Noun | 1145 |
| 14 | work | Verb | 1662 | 34 | work-related | Adj† | 1131 |
| 15 | disease | Noun | 1660 | 35 | care | Noun | 1119 |
| 16 | be | Verb | 1601 | 36 | impact | Noun | 1099 |
| 17 | safety | Noun | 1580 | 37 | physical | Adj† | 1090 |
| 18 | analysis | Noun | 1552 | 38 | pain | Noun | 1050 |
| 19 | stress | Noun | 1511 | 39 | respiratory | Adj† | 1049 |
| 20 | job | Noun | 1480 | 40 | case | Noun | 1040 |

*POS: part of speech, †Adj: adjective

Supplementary Table S2. Co-occurrence among subcategories

| No | Subcategory | 1 | 2 | 3 | 4 | 5 | 6 | 7 | 8 | 9 | 10 | 11 | 12 | 13 | 14 | 15 | 16 | 17 |
|---|---|---|---|---|---|---|---|---|---|---|---|---|---|---|---|---|---|---|
| 1 | Occupations | 1 | 0.04 | 0.15 | 0.09 | 0.12 | 0.07 | 0.08 | 0.06 | 0.04 | 0.04 | 0.03 | 0.13 | 0.08 | 0.08 | 0.09 | 0.07 | 0.08 |
| 2 | Countries | 0.04 | 1 | 0.05 | 0.02 | 0.03 | 0.02 | 0.04 | 0.04 | 0.02 | 0.03 | 0.01 | 0.03 | 0.03 | 0.03 | 0.04 | 0.02 | 0.02 |
| 3 | Non-intervention | 0.15 | 0.05 | 1 | 0.06 | 0.12 | 0.06 | 0.07 | 0.05 | 0.05 | 0.03 | 0.02 | 0.12 | 0.09 | 0.13 | 0.10 | 0.06 | 0.05 |
| 4 | Intervention | 0.09 | 0.02 | 0.06 | 1 | 0.04 | 0.03 | 0.06 | 0.04 | 0.04 | 0.02 | 0.06 | 0.09 | 0.04 | 0.03 | 0.05 | 0.08 | 0.06 |
| 5 | Risk | 0.12 | 0.03 | 0.12 | 0.04 | 1 | 0.17 | 0.04 | 0.04 | 0.08 | 0.04 | 0.01 | 0.06 | 0.13 | 0.12 | 0.06 | 0.03 | 0.03 |
| 6 | Chemical factors | 0.07 | 0.02 | 0.06 | 0.03 | 0.17 | 1 | 0.03 | 0.02 | 0.02 | 0.05 | 0.00 | 0.03 | 0.11 | 0.07 | 0.03 | 0.02 | 0.01 |
| 7 | Psychosocial factors | 0.08 | 0.04 | 0.07 | 0.06 | 0.04 | 0.03 | 1 | 0.07 | 0.06 | 0.03 | 0.01 | 0.07 | 0.05 | 0.03 | 0.08 | 0.07 | 0.03 |
| 8 | Lifestyle factors | 0.06 | 0.04 | 0.05 | 0.04 | 0.04 | 0.02 | 0.07 | 1 | 0.09 | 0.02 | 0.01 | 0.04 | 0.05 | 0.03 | 0.05 | 0.09 | 0.03 |
| 9 | Physical factors | 0.04 | 0.02 | 0.05 | 0.04 | 0.08 | 0.02 | 0.06 | 0.09 | 1 | 0.02 | 0.02 | 0.04 | 0.04 | 0.02 | 0.07 | 0.05 | 0.02 |
| 10 | Biological factors | 0.04 | 0.03 | 0.03 | 0.02 | 0.04 | 0.05 | 0.03 | 0.02 | 0.02 | 1 | 0.01 | 0.02 | 0.03 | 0.01 | 0.02 | 0.02 | 0.01 |
| 11 | Ergonomic factors | 0.03 | 0.01 | 0.02 | 0.06 | 0.01 | 0.00 | 0.01 | 0.01 | 0.02 | 0.01 | 1 | 0.02 | 0.03 | 0.00 | 0.02 | 0.02 | 0.01 |
| 12 | Therapy and care | 0.13 | 0.03 | 0.12 | 0.09 | 0.06 | 0.03 | 0.07 | 0.04 | 0.04 | 0.02 | 0.02 | 1 | 0.10 | 0.09 | 0.08 | 0.07 | 0.07 |
| 13 | Organ damage | 0.08 | 0.03 | 0.09 | 0.04 | 0.13 | 0.11 | 0.05 | 0.05 | 0.04 | 0.03 | 0.03 | 0.10 | 1 | 0.13 | 0.10 | 0.03 | 0.02 |
| 14 | Disease | 0.08 | 0.03 | 0.13 | 0.03 | 0.12 | 0.07 | 0.03 | 0.03 | 0.02 | 0.01 | 0.00 | 0.09 | 0.13 | 1 | 0.07 | 0.02 | 0.02 |
| 15 | Symptoms | 0.09 | 0.04 | 0.10 | 0.05 | 0.06 | 0.03 | 0.08 | 0.05 | 0.07 | 0.02 | 0.02 | 0.08 | 0.10 | 0.07 | 1 | 0.08 | 0.02 |
| 16 | Productivity | 0.07 | 0.02 | 0.06 | 0.08 | 0.03 | 0.02 | 0.07 | 0.09 | 0.05 | 0.02 | 0.02 | 0.07 | 0.03 | 0.02 | 0.08 | 1 | 0.04 |
| 17 | Safety | 0.08 | 0.02 | 0.05 | 0.06 | 0.03 | 0.01 | 0.03 | 0.03 | 0.02 | 0.01 | 0.01 | 0.07 | 0.02 | 0.02 | 0.02 | 0.04 | 1 |

Numbers in the heat map indicate Jaccard values. The Jaccard value between A and B value was calculated as the number of titles containing A and B, divided by the number of titles containing A or B. The numbers indicate the strength of the relationship between subcategories in the titles. To make the color contrast easy to distinguish, one was not colored.
